# Navigating decision space: Causal structure improves performance in a branching choice task

**DOI:** 10.1371/journal.pone.0336899

**Published:** 2025-12-01

**Authors:** Andreas Arslan, Jonathan F. Kominsky

**Affiliations:** Department of Cognitive Science, Central European University PU, Vienna, Austria; RIKEN CBS: RIKEN Noshinkei Kagaku Kenkyu Center, JAPAN

## Abstract

Previous research has shown that the causal structure of events influences how well they are recalled in episodic memory later on. Here, we aimed to investigate whether these effects apply not only to events that are passively observed but also situations directly shaped by an individual’s decisions. We designed a task in which participants had to traverse decision trees of varying causal structure: ‘Coherent’ trees where each decision followed from the consequences of the preceding decision, and ‘fragmented’ trees where each subsequent decision was only statistically (but not causally) contingent on the preceding decision. In a between-subjects experiment, participants first completed an exploration phase in which they had to explore the decision trees without a specific goal; in a subsequent search phase, they had to reach a target outcome in as few attempts as possible. Analyses of participants’ performance showed that those in the coherent group required significantly fewer attempts to reach a correct outcome than those in the fragmented group. A follow-up experiment surprisingly found that the advantage of causal structure does not depend on episodic memory: Removing the exploration phase barely diminished the positive effect causal coherence had on participants’ performance. In further follow-up experiments without an exploration phase, neither the additional removal of ‘process images’ that show how a choice leads to an outcome, nor the removal of text labels describing decisions, was individually sufficient to equalize performances. Only when both were eliminated at once did participants perform equally well on coherent and fragmented trees. This indicates that cues relating to causal mechanisms (images) and predictive cues (text) each facilitate goal-directed decision making without relying on extensive learning, and that only the absence of both is sufficient to suppress the advantage causal structure provides.

## Introduction

Much of what we experience is a result of the decisions we make. Where we look influences what we see, where we go determines what situations we find ourselves in, and how we behave affects how others treat us in turn. Experiences accordingly do not simply happen to us but are produced by a complex pattern of decisions that range from the immediate motoric (e.g., where we decide to look) to the long-term strategic (e.g., the career path we chose). Not all decisions, however, have equally foreseeable outcomes. Sometimes it may be difficult to discern whether an action has a notable consequence at all or when to expect it. This raises the question: Does the relationship between decision and outcome affect how well we remember what decisions we have made in the past? Here, we ask if decisions with inexplicable, non sequitur consequences are remembered differently than those we can interpret as the *cause* in a cause-effect relationship. Do we have more trouble recalling actions we took if we are unable to link them causally to their outcomes?

Remembering decisions made in past situations is something bound to frequently involve episodic memory, a subsystem of declarative memory understood to be responsible for the representation of individuals’ first-hand experiences [1,2]. Whether the causal structure of events influences the episodic memories they give rise to has been investigated in previous studies [3–13], but most of them manipulated a fixed textual narrative or video which participants only read or observed. Here, by contrast, we aimed to understand how causal structure affects the memories of events that are shaped by individuals’ *explicit decisions*. Instead of studying memories that are the product of passive observation, we intended to devise a task that would allow us to probe the recall of events whose content is determined by the interventions participants choose to make.

There are reasons to believe that the mind should usually be more incentivized to record the outcomes of one’s *own* actions in memory than to track cause-effect relationships in arbitrary observed events. The perhaps most obvious argument supporting this assumption is that events whose evolution and outcome we directly affect are also more likely to reciprocally affect ourselves. These consequences may be immediate and unambiguous (such as reward/punishment or positively/negatively valenced outcomes [14]) but can also have long-term effects on our physical environment, or our social world [15].

### Decision trees and the potential advantage of causal coherence

**Fig 1:**
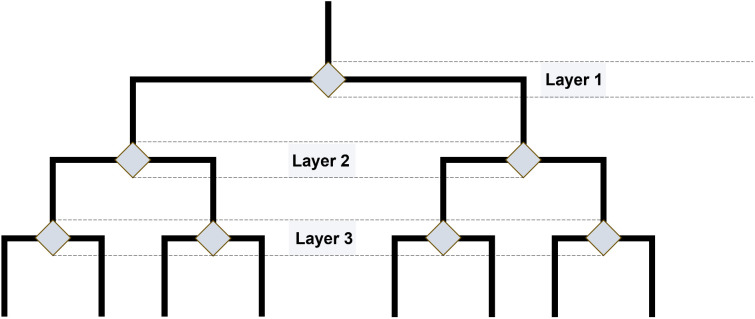
Schematic overview of the decision tree used in all experiments. Gray diamonds indicate decision nodes. The tree begins with the single decision node on layer 1 and ends in 8 different leaves or outcomes.

To test whether the presence of an identifiable causal relationship between decisions and outcomes affects how well an event is remembered, one has to find a way of manipulating the causality of said relationships (i.e., turn it ‘on’ or ‘off’). The general format of a ‘decision tree’ seemed a suitable operationalization in that regard. For the purposes of this paper, a decision tree is simply a series of binary choices leading to unique outcomes: Starting at a single node at which an agent may choose between two available actions, they branch outward, potentially spanning multiple intermediate levels. In our depiction of these trees each choice is a node which is connected to an outcome by an edge (Fig 1). A unique path through such a decision tree, stretching from the starting point to one of its terminal outcomes, corresponds to a specific ‘episode.’ This simple format therefore captures the intuition that some of our experiences can be viewed as the history of our successively made choices and their outcomes.

We can investigate the influence of causal structure on episodic memory by comparing episodes that unfold in *coherent* decision trees (causality ‘turned on’) with those that occur in *fragmented* (causality ‘turned off’) ones. We call an episode coherent whenever it can be interpreted as an unbroken succession of causes and effects. If there are gaps or discontinuities that do not allow for a causal explanation, we term the episode fragmented. The difference between coherent and fragmented stimuli parallels that between a full-length movie and a clip show: Both consist of separate, distinguishable scenes, but while the movie likely features plenty of cause-effect relations that connect what happened early on and what occurs towards the end, the events shown in the clip show are causally isolated from each other.

Precisely because decisions and outcomes in coherent decision trees are linked by causal relations, we suspect that storing such decision-outcome pairs in the form of episodic memories is adaptive in that they contain knowledge that is applicable beyond the specific situation. Once a cause-effect relation between a decision and an outcome is identified and committed to memory, it may be useful in guiding one’s actions in similar future situations. By contrast, the very arbitrariness of how decisions map onto outcomes in fragmented decision trees is a cue that these relations are specific and do not generalize to other circumstances. A fragmented decision tree has to be exhaustively explored to be mapped. Conversely, in a coherent decision tree it may often be possible to *predict* approximate outcomes even before taking action. Indeed, when confronted with a binary choice like ‘Burn the evidence OR shred the evidence’, it does not take much mental effort or specific knowledge to foresee that burning the evidence will create smoke and ash but is a more thorough means of destroying it than putting it in a paper shredder. This may be relevant to consider in cases where there are additional constraints such as ‘make sure to not get caught’ or ‘make sure not a single letter remains legible.’ When there is no inferable causal relation between decision and consequence, however, prediction becomes nearly impossible.

Relatedly, in a causally coherent environment the outcomes of decisions can provide agents with information about what *might have happened* had they acted differently. This is again especially pertinent if an agent’s successive actions are in pursuit of a particular goal. For instance, in the process of trying to fix a broken device, following certain interventions (tightening a screw, cutting a wire) it may be possible to tell whether they have partially restored its functionality or actually have made things worse. This enables an agent to reason counterfactually [16] and may occasionally compel them to reverse an action and do something else in future attempts. In fragmented decision trees, there is no reliable notion of proximity to a desired state, and paths (sequences of decisions) have to simply be memorized.

### Present experiments

Based on the preceding considerations, we hypothesized that people would remember events they experience in a coherent decision tree better than those they experienced in a fragmented one. To test our hypothesis, we designed a novel branching choice task. In it, participants had to make three successive decisions about how to combine various everyday items. Depending on their choices, they would see different outcomes. All experiments we conducted followed a between-subjects design, in which participants were assigned to either a coherent or fragmented condition. Those in the latter group had to complete a version of the task in which discontinuities between decisions and outcomes made it impossible to infer a mechanistic causal relation between them.

As we set out to study the impact of causal structure on people’s episodic *memory* of the decisions they make, Experiment 1 was split into an exploration and a recall phase. In the first phase, participants were simply asked to make whatever choices they wanted until they had reached four different outcomes. Later, in the recall phase, they were shown an outcome they had seen before and were tasked with recreating this outcome, which required them to remember precisely which sequence of choices maps onto each outcome and act accordingly. To foreshadow, Experiment 1 indeed showed that participants in the coherent group required fewer attempts to reach a target outcome in the recall phase.

To establish whether the effect we detected was attributable to episodic memory, we ran Experiment 2, which in essence was Experiment 1 without an exploration phase. Participants had to reach a never-before-seen outcome by means of trial and error. We found that even without previous exposure to a decision tree, people were considerably better at navigating from a starting point to a target outcome if the decision tree was causally coherent. Apparently, the way the choices were presented frequently enabled participants to accurately predict their outcome. In Experiments 3 and 4, we removed various cues from the task to assess which of them might be most strongly linked to the advantage causal structure affords. Finally, Experiment 5 was conducted to rule out that the visual discontinuities themselves that go hand in hand with causally fragmented event structures might be responsible for the performance differences we repeatedly found. Its results suggest this discontinuity alone does not affect performance. Causal structure itself therefore appears to exert a direct, consistent influence on performance, but this effect does not primarily depend, in this case at least, on retrospective episodic memory.

## Experiment 1

Experiment 1 aimed to investigate whether causally coherent decision-outcome sequences are better remembered than causally fragmented ones. We call a decision tree coherent if all state changes (for example, a previously straight teaspoon being bent) that might occur within it are the result of a clearly identifiable effect. This is not the case for fragmented decision trees, where state changes can take place in the absence of causes (the bent spoon is suddenly straight again). We hypothesized that causal coherence facilitates learning which decisions map onto which outcomes: People who interact with a coherent decision tree should be better able to learn what choices lead to a particular outcome. Fragmented decision trees are just as deterministic as coherent ones, but their state transitions do not abide by known causal relations. Based on this, we predicted that it would be more difficult for people to remember the paths through them that connect their starting and end points.

To test the potential influence of causal coherence on episodic memories of choice-contingent events, we designed an interactive task whose basic structure corresponded to a decision tree with seven nodes distributed across three layers (Fig 1). Each node was connected by two edges to two nodes in a downstream layer. The nodes mark the points where participants had to make a binary decision.

**Fig 2:**
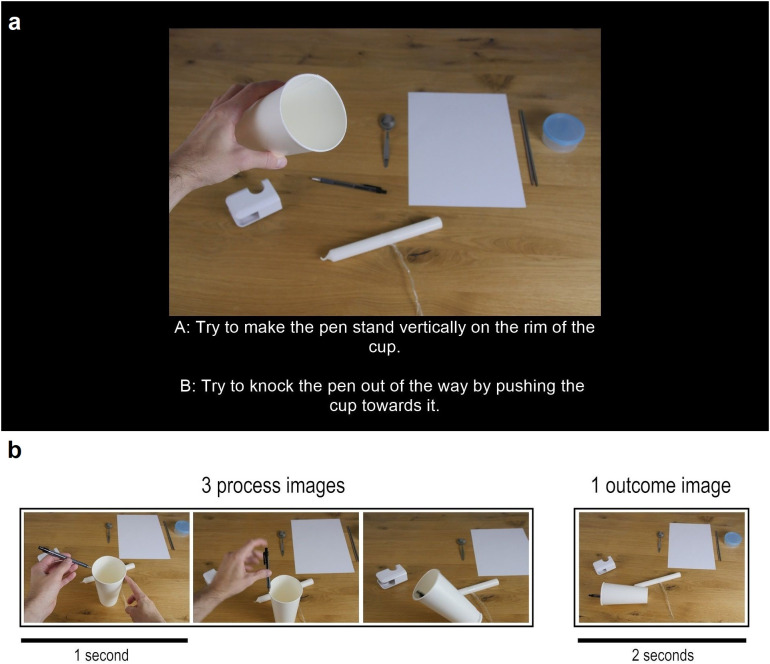
Image types. **(a)** Example of a *decision image* and accompanying *decision text* as it was presented to participants. They had to press A or B on their keyboard to advance. **(b)** The four photographs consecutively shown after each decision made by a participant. Three *process images*, each lasting for 1 second, depicted how an action physically unfolded. The *outcome image* followed immediately after and was shown for 2 seconds.

The task itself was about using and combining a number of everyday objects (such as pens, candles, or knitting needles) in frequently unusual ways. Participants would have to make a choice about what to do with certain objects (‘Try to make the pen stand vertically on the rim of the cup.’) and would then watch the consequences of this choice unfold. Traversing a decision tree from its beginning to one of its eight endpoints (or ‘leaves’) required three decisions. At each node participants saw a photograph (the *decision image*) displaying an arrangement of eight objects and two options, A and B, below the image (Fig 2). Selecting either A or B would trigger the presentation of three *process images* and, finally, an *outcome image*. The outcome image would be followed by another decision node or, if the participant had already been on the third layer of the tree, the end of that particular trial.

**Fig 3:**
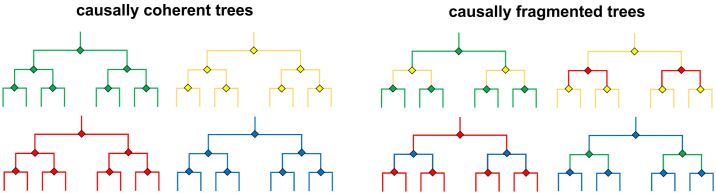
Coherent vs fragmented decision trees. Fragmented decision trees were created by rearranging coherent ones (see color coding).

We created both causally coherent and causally fragmented trees. In the former, the consequences of decisions participants made at previous nodes always carried over to the present one. If, for example, an earlier decision led to a piece of paper being torn, it would remain in that state throughout the episode. As we had created four different coherent trees that all featured the same set of eight objects, we could generate fragmented trees by replacing the second layer of one tree with that of another (Fig 3). In this way, a causal disconnect between the outcome shown in layer one and the image shown at the decision node in layer two was introduced. At the same time, a similar discontinuity was introduced between the outcome of the extraneous layer two and the image shown at the decision node in layer three. In other words, the first two decisions made in a fragmented tree would always result in a clear causal disconnect. Using the exact same set of images in the coherent and fragmented trees allowed us to rule out that potential performance differences could be the result of low-level stimulus properties we had not accounted for. It necessitated, however, a between-subjects experimental design, because the same photos could not be used in both coherent and fragmented trees without adding unwanted additional complexity and ambiguity. We also avoided simply exchanging the second layers of two trees, as participants may have noticed this and interpreted it not as a causal discontinuity but rather a ‘misplaced’ layer (Fig 3 illustrates that layer changes were never *reciprocal*; for example, the second layer of the mostly green fragmented tree on the top left comes from the yellow coherent tree, but the mostly yellow fragmented tree on the top right has a second layer that comes from the red coherent tree).

To measure the differential effect of causal structure on episodic memory, we assigned participants to either a coherent or fragmented group. In the online experiment they first had to complete an exploration/familiarization phase, in which they had the chance to interact with the (either fragmented or coherent) decision trees and thereby learn which decisions lead to which outcomes. This was followed by a task phase, where they were asked to reach a randomly selected target outcome they already had seen previously. Our preregistered hypothesis (https://osf.io/rgfu6/?view_only=2944b5fd4d42eb8fe560b83919d592) was that participants in the coherent condition would require *fewer attempts to reach a target outcome*.

### Method

#### Participants.

Forty adult participants took part in Experiment 1 (N = 20 in the coherent and N = 20 in the fragmented group). Participants were recruited via Prolific (https://www.prolific.com) and received £4.50 in compensation. We did not focus on any specific group or demographic, nor did we place any geographical constraints on the pool of participants. The only prerequisites we asked for were fluency in the English language and no previous participation in pilot studies associated with the experiment. Prospective participants gave informed consent via a Qualtrics survey (https://www.qualtrics.com) and then completed the actual online experiment on the Pavlovia platform [17]. Data collection for this experiment took place on December 21^st^, 2023 (as all reported experiments were conducted online, the recruitment period and data collection coincided).

#### Ethical considerations.

This and all subsequent experiments were approved by CEU’s Psychological Research Ethics Board (Reference number: 2022/17). All procedures performed in this study were in accordance with the ethical standards of the 1964 Helsinki Declaration and its later amendments or comparable ethical standards. Informed consent was obtained from all participants in the aforementioned Qualtrics survey before they interacted with any experimental stimuli of Experiments 1–5. All analyses for all experiments described here were conducted on anonymized data that contain no identifying information.

#### Materials and procedure.

For this experiment, we designed a game-like, interactive task using the PsychoPy software package [18]. The PsychoPy scripts, anonymized data, photos and other material relevant to this and the following experiments can all be found in the OSF repository for this study (https://osf.io/jyz8r/?view_only=ff429a798ccd49a1bb84c68649f9ea83).

At its heart, the task consisted in navigating through a branching, symmetrical tree structure and participants’ input determined what visual and textual stimuli would be presented to them. We created four coherent and four fragmented decision trees (Fig 3), which, continuing with the game analogy, could be viewed as different ‘levels.’ The underlying mechanics of the task were the same for all trees while the specific content (images, text, available decisions) varied. Each tree comprised 63 unique photographs (amounting to a total of 252 for all four) and seven pairs of sentences describing the choices available to participants at a given decision node. Structurally, all the trees were identical: They consisted of seven nodes distributed across three layers (one on the first, two on the second, four on the third). At each node, the sentence pair below the photograph described the two options between which participants could choose. Each tree had eight outcomes.

Every photograph in a tree belonged to one of three categories: decision image, process image, or outcome image. Decision images (Fig 2, a) were the aforementioned photographs presented at nodes of the tree (together with text explaining what actions can be taken at this point). They always featured an array of objects in the background and a hand (or two) holding one or two objects in the foreground. The objects in the background were always arranged in the same order from left to right, with gaps in the lineup where the object(s) currently held by the hand would have been. Process images were sets of three sequentially presented images that were shown on screen after a choice had been made. They were more tightly framed and often included only objects involved in a current action (Fig 2, b). Generally speaking, they were dynamic images depicting the unfolding outcome of a previously selected action. The consecutive presentation of three process images (each was shown for 1 second) was always followed by an outcome image. Outcome images, like decision images, were framed in a manner that ensured the complete configuration of objects (the whole ‘scene’) was visible and were shown for 2 seconds. The presentation of an outcome image in the third layer of a tree also marked that one of its endpoints has been reached.

All trees featured the same set of eight everyday objects: A candle, a cup, a pair of knitting needles, a pen, a piece of paper, a plastic container, a spoon, and a tape dispenser. The first decision image in a tree depicted two hands, each holding an object and the remaining six items arranged in the background. Subsequent decision images showed one or two hands holding only a single object. Trees did not differ in regard to which objects were present but in how they could be combined. For example, while in one tree participants were given the option to try to balance a ballpoint pen on the rim of a paper cup, in another there was the option to use the pen to poke a hole into a wax candle. Importantly, the actions available to participants did not necessarily ‘succeed’: Around half of the time, things would go awry (such as the pen immediately tipping over). As the ways of combining the objects were different in all of the trees, the overall ‘scenes’ (the placement and state of *all* the objects) resulting from the participants’ consecutive decisions were also distinct.

In coherent decision trees, the decision images presented in layers two and three were causally compatible with the outcome images that preceded them. For example, if an outcome image showed a crushed paper cup, that same paper cup would still be crushed in the decision image following it. In fragmented trees this was not the case: the decision images in layers two and three lacked a discernible causal link to the outcome images that came before. The same 252 photographs were used for both the coherent and fragmented trees. This was possible because we created fragmented trees by combining two coherent ones (Fig 3). Specifically, we switched out the second layer of each coherent tree with that of another one. In this fashion, two causal discontinuities were introduced: An incoming one (the outcome of layer one is not compatible with the decision image shown at the beginning of the incongruous layer two) and an outgoing one (the outcome of layer two is again incompatible with the decision image at the beginning of layer three). To avoid potential confounds arising from this shuffling around of layers, we systematically controlled and varied which objects appeared how often and on which layers in coherent trees, and also what objects they appeared with together. Thus, the fragmented trees were identical to coherent ones regarding the frequency with which objects were shown and with respect to which could be combined on each layer. The only difference was that their discontinuous structure discouraged the inference of causal connections across layers.

The experiment itself was split into an exploration and a task phase. During the exploration phase participants were asked to explore each of the four trees. The only instruction given to them was that they had to reach four unique outcomes (i.e., half of the total eight) of each tree presented to them. Whenever they had made three decisions and arrived at a terminal leaf of the current tree, a counter informed them of how many unique outcomes they had already discovered. Once they reached four, they could proceed to the next tree. As this was a between-subjects experiment, participants either interacted with only coherent or only fragmented trees. The order in which trees appeared in both the exploration and task phase was randomized.

In the task phase, for each of the four trees one of the outcomes they had discovered earlier was randomly selected to be the *target outcome*. Participants now had to interact with the same trees they already knew from the familiarization phase but were instructed to make the decisions that would lead them to the target outcome. This was conveyed by showing them the outcome image corresponding to the target outcome and a text instruction below the photograph. The target outcome was presented only once for each tree, prior to the first attempt. If a participant reached the correct outcome, they could advance to the next tree. If they made one or more incorrect choices and ended up at the wrong leaf of a decision tree, they had to try again, starting at the beginning. Whenever they reached an outcome, a counter informed them of how many attempts they had already made. After successfully navigating to the target outcome of each tree, the experiment concluded. The dependent variable of central interest to us was the average number of attempts a participant required to reach a target outcome.

### Results and discussion

**Fig 4:**
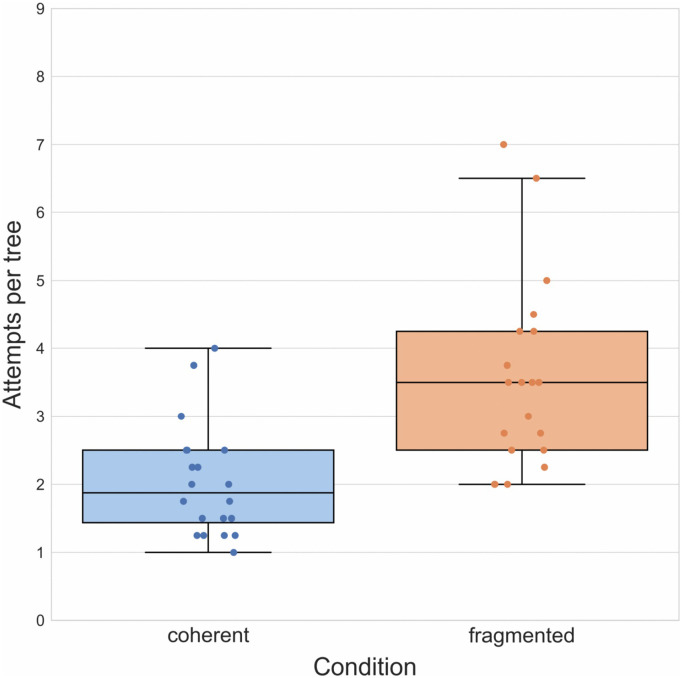
Results of Experiment 1. Boxplots illustrating mean number of attempts required to reach a target outcome in both groups. Boxplot center lines represent the group median.

To test whether causal structure influences performance in this task, we compared the mean number of attempts per tree participants required to reach a target outcome in the coherent group (*M* = 2.03, *SD *= 1.20) with that in the fragmented group (*M* = 3.55, *SD *= 2.86) in an independent samples *t*-test making use of the corresponding function available in the *scipy* Python library [19] and the *pingouin* library to calculate effect sizes [20]. In accordance with our hypothesis, we found that those in the coherent group on average took significantly fewer attempts, *t*(38) = − 4.15, *p* < 0.001, *d* = − 1.31, to arrive at a target outcome (Fig 4).

We also tested whether the groups differed with respect to how frequently participants reached the target outcome on the first try. Here the relevant data were not the average number of attempts but a binary value indicating for each tree whether a participant had managed to find the target outcome in one attempt or not. We conducted a *χ*^2^-test to compare the proportion of ‘single-attempt successes’ in the two groups and found a significant difference, *χ*^2^(1, N = 40) = 5.58, *p* = 0.018, with 41.2% of trials in the coherent group versus 23.7% in the fragmented group being successful first attempts. Noting this high number of single-attempt successes, we performed an exploratory reanalysis of the data to test whether they were the sole driver of the superior performance in the coherent group. We removed the one-shot successes from the dataset and calculated the mean number of attempts from the values that remained (only a single participant had to be removed because they succeeded on the first try in all four trees). We subsequently conducted another *t*-test comparing attempts in the coherent (*M* = 2.60, *SD* = 0.67) and fragmented group (*M* = 4.41, *SD* = 1.66), finding that performances still differed significantly, *t*(37) = − 4.32, *p* < 0.001, *d* = − 1.38. This was the only analysis in Experiment 1 we had not preregistered.

To detect potential item effects, we conducted a 2 (Condition) x 4 (Tree) mixed ANOVA for this and all subsequent experiments, which can be found in the supplemental information (S1 File). To summarize, these analyses revealed no significant item effect or interaction in any of the five experiments.

The pattern of results was in line with our preregistered hypothesis that the causal structure of choice-contingent dynamic events influences how well they are remembered and, specifically, that events with coherent causal structure are remembered better. A natural way of interpreting the findings is to assume that participants used the episodic memories they had formed while exploring the decision trees during the familiarization phase to guide their choices in the task phase. Experiment 1 alone, however, does not definitively show that participants mainly relied on memory while completing the task. It is possible that predictive processes played a role as well: People may have used the decision text to envisage what the arrangement on the table might look like after selecting A or B and then picked the one that was more similar to or compatible with the target outcome shown to them.

## Experiment 2

While Experiment 1 yielded results that aligned with our hypothesis that the causal structure of choice-contingent dynamic events affects episodic memories of these same events, our design did not directly test the role of memory. Experiment 2 sought to determine if they were relying on memory (vs. prediction), by removing the exploration/familiarization phase. This baseline performance would allow us to get a better sense of the relative contributions of episodic memory on the one hand and predictive or inferential processes on the other.

The removal of the exploration/familiarization phase was sufficient to turn the memory task into a prediction task. Participants were shown a target outcome (an image of objects arranged on a table) as before and instructed to reach it, but now they had to rely on guessing and conjecture rather than memory to get there. Fragmented decision trees offer very little in terms of clues that might aid prediction and therefore all but force participants to rely on trial and error. Matters are not that straightforward regarding coherent decision trees. Although we purposely phrased the decision text at each node in a way that ensured that it would not be a *reliable* predictor (around half of the time the action undertaken following the decision ‘fails’ or has consequences the text does not foreshadow) it still provides approximate information about what is going to happen. For example, if there is the option to ‘stab the plastic container with the knitting needles,’ one may not be able to confidently predict whether the needles penetrate the container but might still reasonably suspect that one or more of the objects involved will get damaged (and also have an intuition about what type of damage to expect). In conjunction with the target outcome, participants could engage in a kind of hypothetical reasoning to determine the best possible choice. Although very different in execution, this kind of task structure bears some resemblance to classic studies that looked at how people evaluate situations and the causal impact of choices by comparing them to alternatives [21]. A similar kind of “simulation-based” [22] reasoning could arguably be applied when trying to bring about a target outcome in a coherent decision tree, both prospectively (hypothetical reasoning) and retrospectively if one reaches a non-target outcome (counterfactual reasoning). Whenever a participant has to make a decision, they may simulate the outcome of each alternative. They then compare this possible simulated reality to the target outcome and check whether they are compatible. If, for example, the decision they are considering will likely lead to a knitting needle being bent or broken but they know that it was perfectly intact in the outcome image they were shown, this might be a strong signal that they should pick the other option. In other words, they would use prediction to *avoid* intermediate outcomes that seem to close off the possibility of ever reaching the target outcome.

If the superior performance on coherent decision trees we saw in Experiment 1 was mainly due to coherent events being *remembered* better, removing the familiarization phase and thus the opportunity to rely on episodic memories of choices previously made should put the coherent and fragmented groups on equal footing. This was indeed our preregistered hypothesis for Experiment 2 (https://osf.io/6nqp7/?view_only=20827bca889e44aab9f3f1381ed1bacd). Conversely, if participants in the coherent group still did better, it would indicate that coherent decision trees are not necessarily more memorable but more *predictable* than their fragmented counterparts.

### Method

#### Participants.

40 participants took part in this experiment (N = 22 in the coherent and N = 18 in the fragmented group; the difference in participant numbers is due to imperfect randomization). The platforms used to recruit participants and host the experiment were the same as in Experiment 1 and identical for all subsequent experiments, as were the requirements for participation. Data collection for this experiment took place on January 15^th^, 2024.

#### Materials and procedure.

Materials and procedure were equivalent to Experiment 1, with the exception that the exploration/familiarization phase was entirely absent. Instead, the target outcome for the test phase was determined by randomly selecting one of the eight possible outcomes. Furthermore, the instructions accompanying the presentation of each target outcome image were modified to fit the changed task structure: Rather than being asked to try to *remember* which choices lead to the target, participants were told to simply *guess*.

### Results and discussion

**Fig 5:**
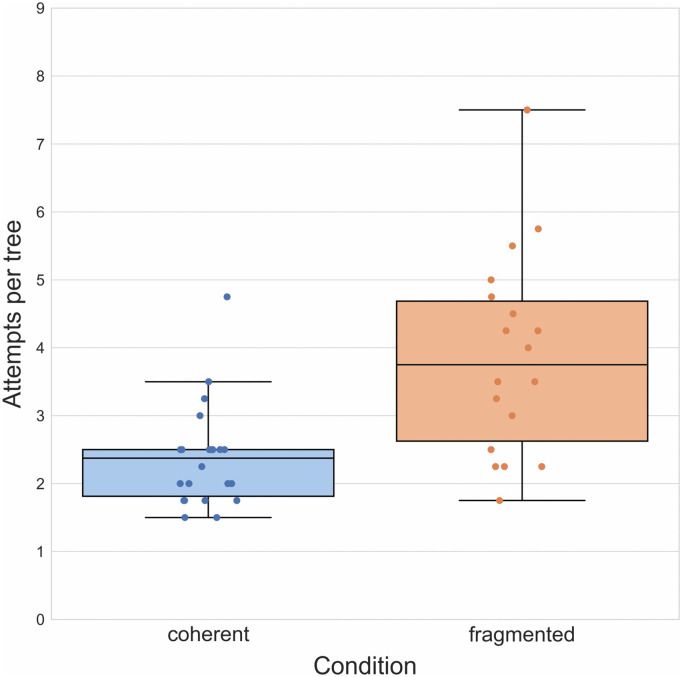
Results of Experiment 2. Boxplots illustrating mean number of attempts required to reach a target outcome in both groups. Boxplot center lines represent the group median.

We repeated the same analyses we conducted in Experiment 1, and to our surprise, found a qualitatively identical and quantitatively very similar pattern of results. Comparing the mean number of attempts necessary to reach a target outcome in the coherent (*M* = 2.37, *SD* = 1.66) and the fragmented group (*M* = 3.87, *SD* = 2.92) using an independent samples *t*-test, *t*(38) = − 4.13, we again found, contrary to our hypothesis, a significant difference, *p* < 0.001, *d* = − 1.31 (Fig 5). Moreover, a *χ*^2^-test comparing the proportion of single-attempt successes in the coherent (40.9%) and fragmented group (20.8%) showed a significant difference between the two, *χ*^2^(1, N = 40) = 7.35, *p* = 0.007. As in Experiment 2, we then removed one-shot successes from the dataset and recalculated the average of attempts (in this case, no participant had performed ‘perfectly’) and repeated the initial *t*-*t*est. A comparison of attempts in the coherent (*M* = 3.44, *SD* = 1.14) and fragmented groups (*M* = 4.78, *SD* = 1.87), showed that performances still differed significantly, *t*(38) = − 2.80, *p* = 0.008, *d* = − 0.89. This was again the only exploratory (i.e., not preregistered) analysis we report here.

Overall, removing the familiarization phase resulted in task performances that were very similar to those seen in the previous version of the experiment. This pattern of results ran counter our preregistered hypotheses: Participants in the coherent group who had no previous exposure to the trees were still able to frequently reach a target outcome on the first try, having been provided with no information beyond an image that shows the configuration of all objects at that desired endpoint. This suggests that the cues provided to participants in the task are in some way more informative in coherent trees and enable them to reach the target more quickly.

## Experiment 3

Experiment 2 demonstrated that superior performance on the branching choice task in the coherent condition is not dependent on *remembering* a sequence of choices that leads to a specific goal but can be achieved by prediction alone. This leaves open the question of what elements of the task *are* indispensable to causal coherence facilitating better performance. When considering the structure of the experiment in schematic terms and disregarding the specific content of each decision tree, there are two broad categories of cues likely to be relevant to how many attempts participants require to successfully complete the task: the *process images* (the three images following the selection of either A or B and showing the actual physical consequences of the decision) and the *decision text* (the textual description of the choices A and B given at each node).

In Experiment 3, we removed the process images to gauge the extent of their contribution to the performance disparity between the coherent and fragmented groups (OSF preregistration: https://osf.io/dg4j3/?view_only=d83e6ef47e9946eb92eb0c4133efb84). Because they illustrate the concrete physical interactions of objects occurring between decision and outcome, they obviously convey important causal information on a comparatively granular, mechanistic level. They therefore might be more informative in the causally coherent condition than the fragmented one, as in the latter the discontinuities between tree layers ensure that the chain of events shown in the process images does not help establish a link between choice and outcome. Seeing that process images are presented only *after* a choice has been made, however, it is unlikely that they are the main factor driving the single-attempt successes that were so prevalent in the previous two experiments in the causal condition. It may nevertheless be the case that they have an ‘orienting effect’ and that their removal (and the concomitant removal of cues helping to represent the causal structure of the overarching event) impairs participants’ ability to effectively guess what the correct path to the target outcome is.

### Method

#### Materials and procedure.

Experiment 3 was identical to Experiment 2, with the sole exception that the three process images previously shown after a participant made a choice were absent. Instead of three photographs, the numbers 3, 2, 1 were presented against a black background (each for a second to match the duration of the images they replaced). An outcome image was subsequently presented for 2 seconds as in earlier experiments. 62 adults participated in this experiment, 2 of which were removed due to exceedingly poor performance, leaving a total of 59 participants (31 in the coherent, 28 in the fragmented group). The exclusion criterion we applied in this and subsequent experiments was lenient: Those who exceeded more than twenty attempts on a single decision tree were excluded, as were those who exceeded ten attempts on at least two trees. Data collection for this experiment took place on February 15^th^, 2024.

### Results and discussion

**Fig 6:**
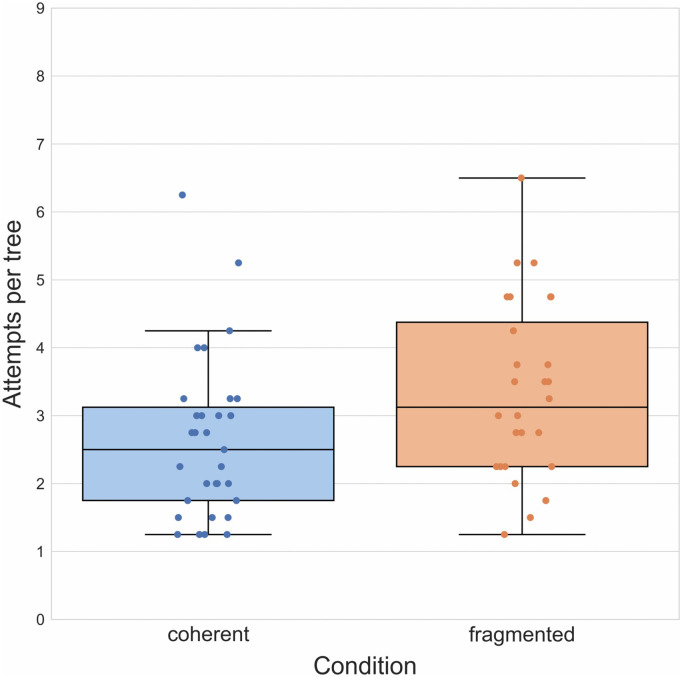
Results of Experiment 3. Boxplots illustrating mean number of attempts required to reach a target outcome in both groups. Boxplot center lines represent the group median.

A *t*-test comparing the mean number of attempts participants required in the coherent (*M* = 2.64, *SD* = 2.13) and fragmented group (*M* = 3.34, *SD* = 2.38) showed a significant difference, *t*(57) = − 2,16, *p* = 0.035, *d* = − 0.56, between the two (Fig 6). We further conducted an exploratory *χ*^2^-test comparing the proportions of single-attempt successes in each group (coherent = 37.1%; fragmented = 24.1%) and found a significant difference, *χ*^2^(1, N = 59) = 4.65, *p* = 0.031). Again in keeping with previous experiments, we removed single-attempt successes and repeated the initial *t*-test, comparing the resultant mean number of attempts of the coherent (*M* = 3.53, *SD* = 1.53) and fragmented group (*M* = 4.00, *SD* = 1.28), but in this case there was no significant difference, *t*(57) = − 1.28, *p* = 0.21, *d* = − 0.33, suggesting that one-shot successes were largely driving the group difference.

Contrary to our preregistered hypothesis, the removal of process images did not result in equal performances of both groups: Participants in the coherent condition still required fewer attempts to reach a target outcome and more frequently did so on the first attempt. The effect size of the *t*-test was however noticeably diminished. As could be suspected from the fact that process images are shown after a choice has been made, their removal did not bring the proportions of single-attempt successes in the coherent group down to the level of the fragmented group. Evidently the mechanistic information process images convey accounts only partially for the better performance in the coherent group. By the same token, this implies that the descriptions of the actions in the decision images are sufficient to drive the observed first-attempt-success difference between the two groups.

## Experiment 4

The purpose of Experiment 4 was to function as a complement to 3 and assess whether removing the decision text describing the available choices would impact performance. Contrary to process images, the decision text accompanying the image at each node is prospective in character (Fig 2). It characterizes a possible action by describing its outcome, for example: ‘Try to open the plastic container using the knitting needles.’ The sentences describing each choice clearly communicate an intention and, in the coherent condition, at least approximately suggest the consequences of a choice (even though, as mentioned earlier, the stimuli used in all experiments were designed such that in half of the cases the process images showed an unsuccessful attempt leading to a result image showing an unintended outcome). It would thus be very unexpected if participants of either group were able to frequently reach the target outcome on the first attempt without the cues the decision texts provide.

### Method

#### Materials and procedure.

Experiment 4, like Experiment 3, was identical to Experiment 2 aside from a small modification: The decision text beneath each image presented at a node of a tree was replaced by the simple instruction ‘Press A or B.’ Participants therefore had to make choices without any prior knowledge about how the available options differ. What kind of action A and B respectively trigger at each node could only be learned by selecting an option at random and committing what follows to memory. 51 adults took part in this experiment, one of which was removed from the analysis due to very poor performance, resulting in a final N = 50 (26 in the coherent, 24 in the fragmented group).

It should be mentioned that we originally preregistered N = 36 as the target sample size for this experiment, as it would have provided us with sufficient power to conduct a planned analysis that aimed to integrate results from two experiments (OSF registration: https://osf.io/6naj9/?view_only=89d9ec1c625e4e10bf3ef0ae9dcffaecs). Upon viewing the findings of all five experiments side-by-side, and seeing how they substantially deviated from our hypotheses, we abandoned this approach and opted to conduct the same straightforward *t*-test comparisons of the number of attempts for all experiments (1–5). As the sample size of N = 38 we effectively arrived at was low compared to the ‘complementary’ Experiment 3’s (N = 60) we pooled our sample of 38 participants with a pilot sample to arrive at N = 51. The individual results for both the N = 38 and the N = 13 pilot sample can be found in the SI. The aforementioned is the *only* pilot we conducted before running what was initially intended to be the ‘full’ experiment 4. Data for this experiment were collected on January 29^th^ and February 5^th^, 2024.

### Results and discussion

**Fig 7:**
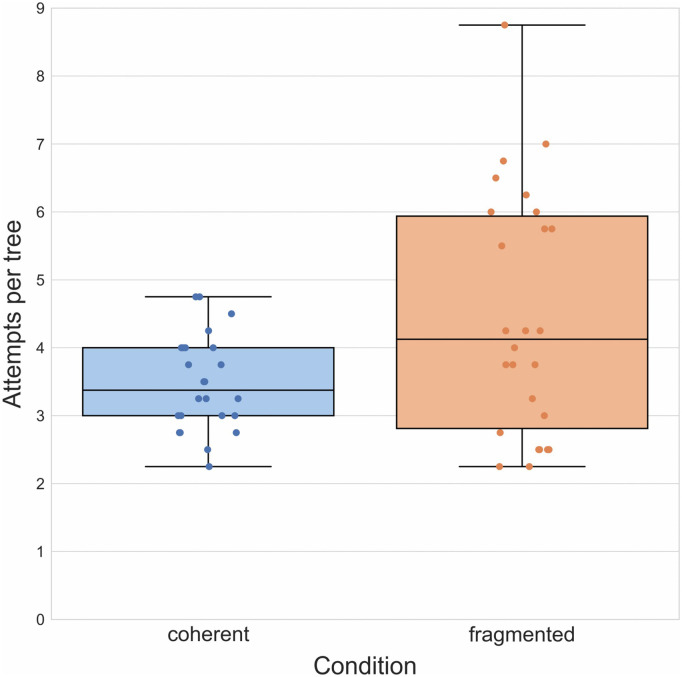
Results of Experiment 4. Boxplots illustrating mean number of attempts required to reach a target outcome in both groups. Boxplot center lines represent the group median.

We again conducted a *t*-test comparing the number of mean attempts required to reach a target outcome in the coherent (*M* = 3.48, *SD* = 2.08) and fragmented group (*M* = 4.45, *SD* = 3.18) finding once more a significant difference between the two, *t*(48) = − 2.49, *p* = 0.016, *d* = − 0.70 (Fig 7). A χ^2^-test checking whether the proportion of single-attempt successes varies with condition (coherent: 14.6%; fragmented: 8.7%) was not significant, *χ*^*2*^(1, N = 50) = 1.72, *p* = 0.199. We nevertheless performed an exploratory *t*-test comparison of group means after single-attempt successes had been removed from the data (coherent: *M* = 4.01, *SD* = 1.09; fragmented: *M* = 4.75, *SD* = 1.74) that narrowly failed to reach significance, *t*(48) = − 1.80, *p* = 0.078, *d* = − 0.51, likely due to the reduced sample size (post-hoc observed power: 55%).

Similar to what we found in Experiment 3 regarding process images, the removal of informative decision texts was not sufficient to eliminate the effect of causal structure on performance in this task. The pattern of results does not completely mirror that of Experiment 3: In accordance with our hypothesis, when deprived of decision texts that offer an approximate prediction of the consequences of a given choice, participants in the coherent group no longer managed to reach the correct outcome on the *first* try more frequently than those in the fragmented group. It should furthermore be noted that participants in both groups performed substantially worse than in Experiment 3, which included the decision text: Both a *t*-test comparing the average number of attempts in the coherent groups of Experiments 3 and 4, *t*(55) = − 3.06, *p* = 0.003, *d* = − 0.83, and an analogous comparison of the fragmented groups of the same experiments, *t*(50) = − 2.62, *p* = 0.011, *d* = − 0.71, were significant.

We additionally tested our preregistered hypothesis that participants in the fragmented group would make more errors on the second layer than participants in the coherent group. A wrong decision on the second layer was only counted as a second-layer error if the previous decision on the first layer had been correct. We suspected that such a performance difference should be particularly noticeable on the second layer of a tree, because it is there that causal fragmentation first becomes apparent. A *t*-test comparing the mean of the total number of second-layer errors in the coherent group (*M* = 3.29, *SD* = 1.43) and the fragmented group (*M* = 4.46, *SD* = 2.80), however, showed no significant difference, *t*(48) = − 1.80, *p* = 0.078, *d* = − 0.51.

Upon inspecting the data, we decided to additionally perform an exploratory analysis of differences between the groups regarding the mean number of errors made on the *third* layer. Again, an error was only classified as a third-layer error if the previous two choices had been correct. We conducted another *t*-test, comparing the coherent (*M* = 1.79, *SD* = 1.04) and fragmented group (*M* = 3.23, *SD* = 1.93), and found that the latter made significantly more third-layer errors, *t*(48) = − 3.18, *p* = 0.003, *d* = − 0.90. It may be relevant to note that this effect was not only significant in the pooled sample but the N = 12 pilot and the N = 38 sample as well (see SI for more statistical information).

## Experiment 5

Experiment 5 functioned as a ‘sanity check’ experiment and was the only one in this paper we did not preregister. It combined the two modifications to the initial design reported on in Experiments 4 and 5 and therefore lacked *both* process images and informative decision texts. We conducted this final experiment to rule out that there was something about the decision and outcome images *themselves* that drove the performance difference between the two groups. In particular the discontinuous ‘jump’ in the fragmented condition from the arrangement shown in the first decision image to a very different arrangement presented in the second image, might itself be sufficient to hamper performance. If that were the case, the intended manipulation of *causal* structure underlying our design would be confounded with an effect potentially arising from the detection of an event boundary [23].

### Method

#### Materials and procedure.

46 adult participants completed a version of the task that featured neither process images (a countdown was shown instead) nor informative decision text (participants were simply told to ‘Press A or B’ at every node). 2 were removed due to poor performance, leaving a total of 44 participants (20 in the coherent, 24 in the fragmented group). Data collection took place on February 1^st^ and February 22^nd^, 2024.

### Results and discussion

**Fig 8:**
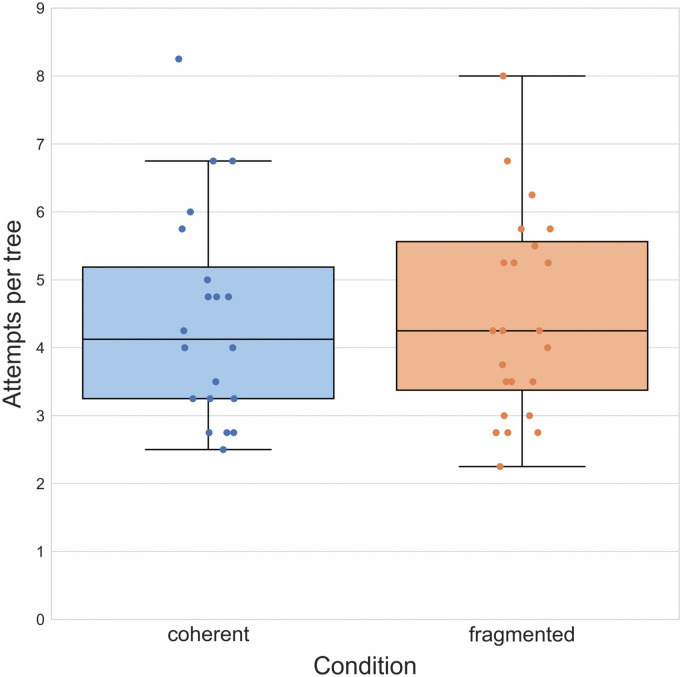
Results of Experiment 5. Boxplots illustrating mean number of attempts required to reach a target outcome in both groups. Boxplot center lines represent the group median.

A *t*-test analysis of average attempts required to reach the target outcome in the coherent (*M* = 4.45, *SD* = 3.17) and fragmented (*M* = 4.63, *SD* = 3.04) groups showed no significant difference, *t*(42) = − 0.35, *p* = 0.73 (Fig 8). We conducted a Bayesian independent-samples *t*-test using JASP [24] which found moderate evidence in favor of the null hypothesis that performance in the two groups was the same, BF_10 _= 0.31. Furthermore, a χ^2^-test checking whether the proportion of single-attempt successes (coherent: 7.5%; fragmented: 11.5%) varies with condition was also not significant, *χ*^*2*^(1, N = 43) = 0.78, *p* = 0.376. The visual mismatch between outcome and decision image in the fragmented group by itself thus has no measurable effect on performance in this branching decision task. Without cues conveying information about the causal structure of these interactive events, participants in both groups perform at equal levels.

## Discussion

Across five experiments that were variations on the same underlying design, we found that the causal structure of events has a considerable effect on participants’ ability to reach a target outcome in a decision tree. Only after both predictive (decision text) and mechanistic cues (process images) had been stripped away did participants in the coherent and fragmented groups perform equally well (Experiment 5). Contrary to our original intentions, the task we had devised did not enable us to get an understanding of the potential relevance of the episodic memory system to the performances we measured. This was particularly well-illustrated by Experiment 2, which lacked an exploration phase that would have allowed participants to encode which combinations of choices lead to which outcomes yet yielded results that were very similar to that of Experiment 1.

While providing no conclusive answer to the question we set out to investigate, these results nonetheless provide some valuable insights. First, participants are surprisingly good at using a target image they have seen only once to effectively reckon their way towards a specific outcome. To succeed at this, the outcome image (a complex configuration of eight objects) has to be held in mind throughout and, at each of the three decision nodes, the choice that will likely bring one closer to the desired leaf of the tree has to be accurately guessed. In Experiments 2 and 3 around 40% of attempts undertaken in the coherent group were successful on the first try. This leaves hardly any doubt about predictive processes being essential to these kinds of one-shot trials. In accordance with that, the removal of specific predictive information in the decision text at each node (participants were instructed to ‘Press A or B’ and not told what either might entail) in Experiment 4 greatly reduced the proportion of single-attempt successes in the coherent group. Nevertheless, overall performance as measured via the number attempts required to reach the target outcome was still better in the coherent group, suggesting that causal coherence does also affect learning, and that the consistent disparity we found is not solely a consequence of fragmented decision trees being less predictable.

Certain aspects of our findings also suggest that the structures of fragmented decision trees, or at least the parts relevant to the completion of the task, are harder to retain or even to learn. One result pointing in this direction is that in Experiment 2 the coherent group’s performance remained superior after removing one-shot trials from analysis. Furthermore, as mentioned above, Experiment 4 provided no obvious predictive cues to participants and those in the coherent group still did better. They required more attempts than in previous experiments, as was to be expected, but so did those in the fragmented group. As the absence of meaningful text labels initially forced participants to press keys at random (there is no way of knowing what choosing either A or B might lead to), the persistence of a performance difference indicates there is a learning effect at play. Interestingly, an analysis of errors made on the third layer, showed a significant difference between conditions: Participants in the fragmented group made more errors on the final layer than participants in the coherent group. Seeing how there was no statistically significant difference between conditions regarding one-shot trials in Experiment 4, this suggests that participants in the fragmented condition more frequently *repeated incorrect choices* at the very end of the decision tree. Put more explicitly, participants in the fragmented group more frequently made two correct choices, failed on the third and final one, and in a later attempt, having again made two correct choices, repeated that error. This can be interpreted as a lack of causal structure leading to a kind of decision amnesia: an incomplete or erroneous recollection of the choices made on previous attempts. Perhaps if the outcome of an action is something entirely unrelated to it, the pairing of decision and consequence is less likely to be properly encoded. While this is an intriguing possibility, an experimental design presenting decision trees with more than three layers to participants likely would be required to comprehensively test this hypothesis. By varying the number of levels of the decision trees, such an extended design would also make it possible to pinpoint a potential ‘transition point’ beyond which participants can no longer mainly rely on prediction. Future experiments that employ larger or more complex decision tree structures therefore may shed light on the possible interplay between prediction and memory driven effects that our findings so far only hint at.

Although the results we report here do not give us the hoped-for insights about how causal structure might influence our memories of decisions we have made in the past, they tell us something about our capacity to anticipate the consequences of our actions. The proportion of single-attempt successes we found in Experiment 2 and even 3 is arguably quite impressive: Participants had to not only keep the outcome image they were shown only once in mind throughout. To make use of it in guiding their decisions, they had to ‘translate’ a photograph of a rather complex array of objects into a mental representation of the target state they were tasked to bring about. In this sense, these findings demonstrate our capacity to engage in sophisticated modal thought to deliberately manipulate and shape our environment – provided sufficient causal information is made available to us.

Many instances of such modal thought can be described as the domain projection of an *anchor* (an object or situation in the present) into the past or future [25]. Both temporal directions would have been relevant to participants as they made their way through coherent decision trees. When first presented with an outcome image, say a plastic container with a knitting needle stuck in it, that photo would have served as the anchor they could project into the past to come up with various candidate states preceding this peculiar arrangement (perhaps someone stabbed the container, perhaps the needles fell from the sky at a high velocity). During the task itself, each available decision could have given rise to a forward projection in time of the arrangement they had in front of them at the present moment (‘If I stab the plastic container with the knitting needle, the container or the needle might get damaged’). If forward domain projection (imagining various consequences of a potential decision) and backward domain projection (imagining various ways the target arrangement could have come about) align, it is a strong cue that one has found a path in the tree that connects the present with the target state. The opposite is also true: If the set of possible future states one imagines will follow a decision is consistently *incompatible* with the target outcome, this clearly suggests that another course of action would be preferable. The experiments we conducted indicate that humans are good at avoiding actions we suspect will prevent rather than lead towards a desired future state. When deprived of reliable cues about cause-effect relations, however, it appears to be much harder to steer clear of dead ends.

## Supporting information

S1 File Additional results. (DOCX)
