# Supplementary material for: Navigating decision space: Causal structure improves performance in a branching choice task
Source: PLoS One. 2025 Dec 1;20(12):e0336899. doi: 10.1371/journal.pone.0336899 (PMC12668605; doi:10.1371/journal.pone.0336899)
Supplement: S1 File — (DOCX) [file pone.0336899.s001.docx]

Supplementary information

Navigating decision space: Causal structure improves performance in a branching choice task

Andreas Arslan^1^ & Jonathan F. Kominsky^1^ ^1^Department of Cognitive Science

Central European University Vienna, Austria

Correspondence: [Arslan_Andreas@phd.ceu.edu](mailto:Arslan_Andreas@phd.ceu.edu)

# Supplementary results

In the interest of brevity and clarity, the discussion in the results sections of the main paper focuses on results relevant to our hypotheses and the theoretical questions we posed. The tables below contain results of additional analyses.

# Testing for stimulus-specific effects: Exp 1 – Exp 5

2 x 4 ANOVAS conducted for all experiments to detect potential stimulus- based effects. Neither the predictor ‘Tree’ (meaning one of 4 decision tree stimuli) nor the interaction ‘Condition x Tree’ reached significance in any of the five experiments. While they did, in some instances, approach ‘marginal significance,’ a look at the performances on the individual decision tree stimuli as illustrated in Supplementary Figures 1 – 5 arguably suggests there is no readily detectable pattern to these performance fluctuations.

**Note:** ‘First tree’ etc. does not refer to the order in which stimuli were presented, but always indicates the same specific stimulus, i.e., ‘Tree1’. The same stimuli were used in all experiments.

**Supplementary table 1.** *Experiment 1. 2x4 ANOVA results.*

| **Predictor** | ***df*** | **MSE** | ***F*** | ***P*** |
| --- | --- | --- | --- | --- |
| Condition (coherent or  fragmented) | 1, 38 | 91.51 | 17.26 | <0.001 |
| Tree | 3, 114 | 10.82 | 2.40 | 0.07 |
| Condition x Tree | 3, 114 | 7.12 | 1.58 | 0.20 |

**Supplementary figure 1.** *Experiment 1. 2x4 ANOVA results.*

*
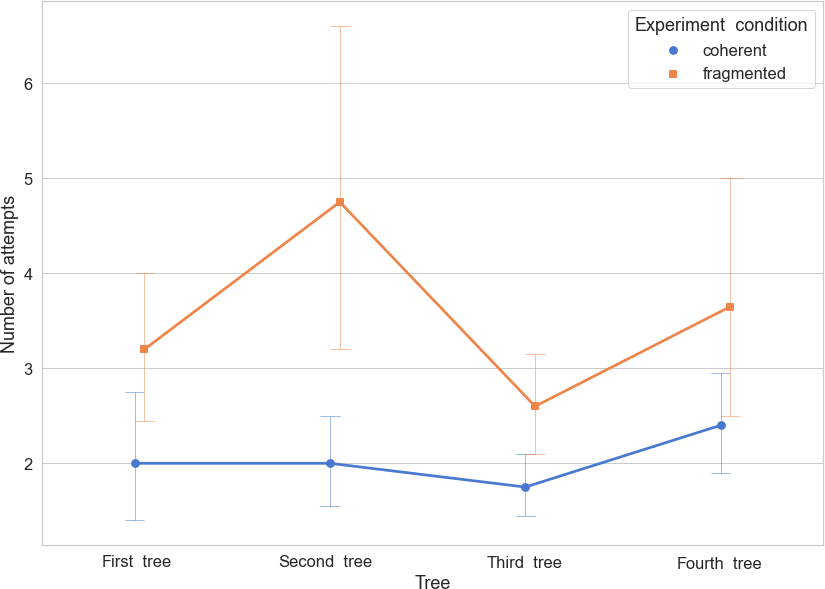
*

**Supplementary table 2.** *Experiment 2. 2x4 ANOVA results.*

| **Predictor** | ***df*** | **MSE** | ***F*** | ***p*** |
| --- | --- | --- | --- | --- |
| Condition (coherent or  fragmented) | 1, 38 | 89.10 | 17.06 | <0.001 |
| Tree | 3, 114 | 0.22 | 0.04 | 0.99 |
| Condition x Tree | 3, 114 | 13.92 | 2.58 | 0.06 |

**Supplementary figure 2.** *Experiment 2. 2x4 ANOVA results.*

*
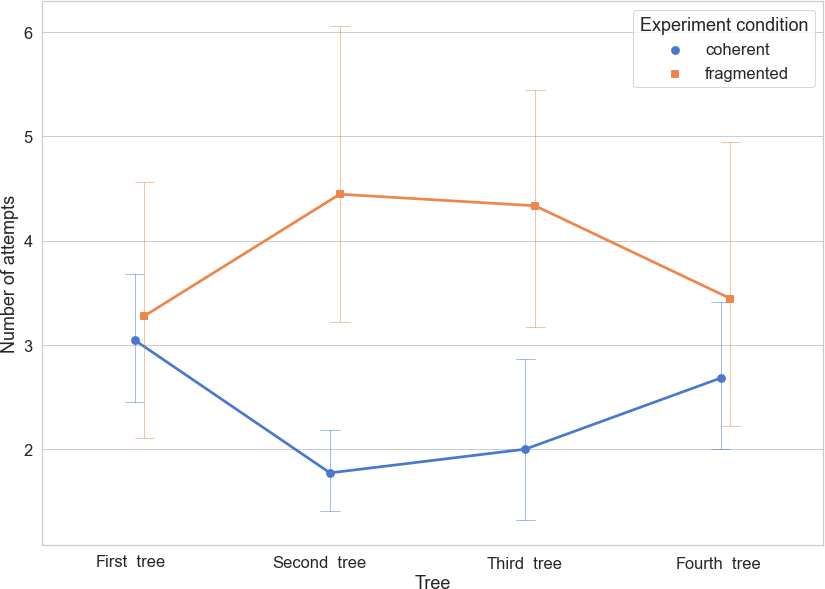
*

**Supplementary table 3.** *Experiment 3. 2x4 ANOVA results.*

| **Predictor** | ***df*** | **MSE** | ***F*** | ***p*** |
| --- | --- | --- | --- | --- |
| Condition (coherent or  fragmented) | 1, 57 | 29.02 | 4.66 | 0.03 |
| Tree | 3, 171 | 8.81 | 1.88 | 0.13 |
| Condition x Tree | 3, 171 | 6.71 | 1.43 | 0.23 |

**Supplementary figure 3.** *Experiment 3. 2x4 ANOVA results.*

*
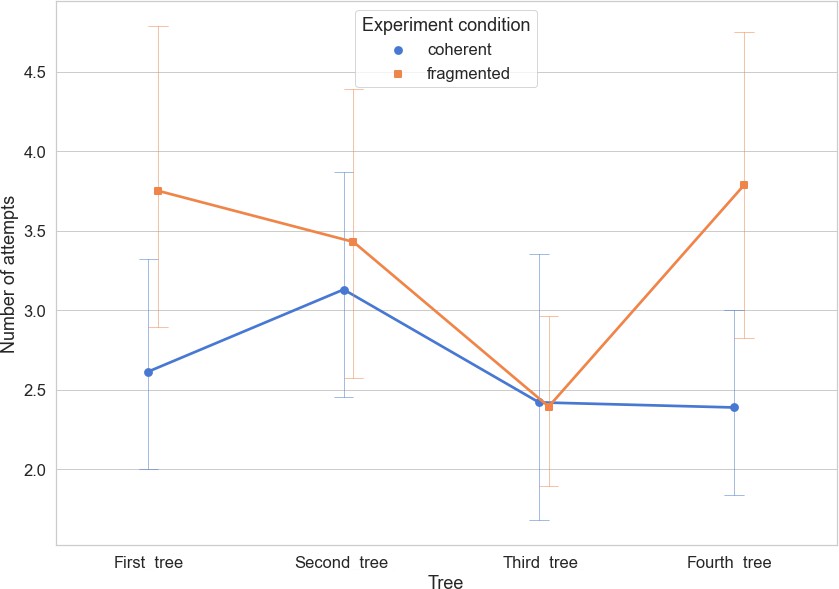
*

**Supplementary table 4.** *Experiment 4. 2x4 ANOVA results.*

| **Predictor** | ***df*** | **MSE** | ***F*** | ***p*** |
| --- | --- | --- | --- | --- |
| Condition (coherent or  fragmented) | 1, 48 | 47.24 | 6.20 | 0.02 |

| Tree | 3, 144 | 1.74 | 0.23 | 0.87 |
| --- | --- | --- | --- | --- |
| Condition x Tree | 3, 144 | 5.55 | 0.74 | 0.53 |

**Supplementary figure 4.** *Experiment 4. 2x4 ANOVA results.*

*
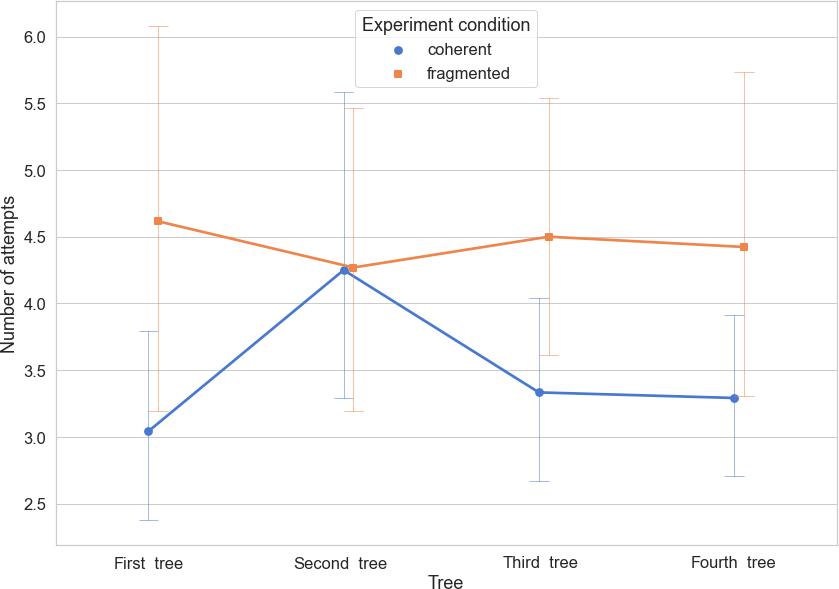
*

**Supplementary table 5.** *Experiment 5. 2x4 ANOVA results.*

| **Predictor** | ***df*** | **MSE** | ***F*** | ***p*** |
| --- | --- | --- | --- | --- |
| Condition (coherent or  fragmented) | 1, 42 | 1.50 | 0.12 | 0.73 |
| Tree | 3, 126 | 10.35 | 1.20 | 0.31 |
| Condition x Tree | 3, 126 | 22.36 | 2.59 | 0.055 |

**Supplementary figure 5.** *Experiment 5. 2x4 ANOVA results.*

*
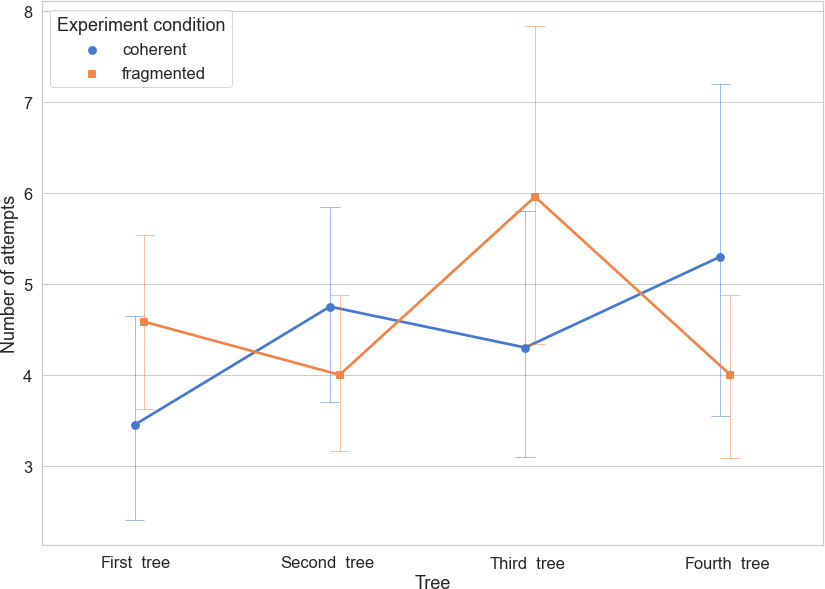
*

# Results of Experiment 4: Individual samples (N=12 and N=38)

As explained in the main body of the paper, the sample of Experiment 4 was obtained by pooling experimental data obtained in two different runs (though under identical conditions). For completeness’s sake, we list the results of some of the central analyses for the subsamples here. Please refer to the paper for additional context.

# N=12 sample (pilot)

Coherent N: 4 Fragmented N: 8

*t*-Test comparison of mean attempts across groups Coherent *M*: 3.75 Coherent *SD*: 0.73

Fragmented *M*: 5.53 Fragmented *SD*: 1.77

*t*(10) = - 1.76, *p* = 0.11, *d* = - 1.08

Chi-squared test for independence (comparison of single-attempt successes)

*χ*^2^(1, N = 12) = 7.71, *p* = 0.005, Cramer’s *V* = 0.40

3^rd^-level errors, *t*-Test comparison

*t*(10) = - 2.30, *p* = 0.04, *d* = - 1.41

# N=38 sample

Fragmented N: 20 Coherent N: 18

*t*-Test comparison of mean attempts across groups Coherent *M*: 3.42 Coherent *SD*: 0.66

Fragmented *M*: 3.97 Fragmented *SD*: 1.52

*t*(36) = - 1.42, *p* = 0.16, *d* = - 0.46

Chi-squared test for independence (comparison of single-attempt successes)

*χ*^2^(1, N = 38) = 1.05, *p* = 0.59, Cramer’s *V* = 0.17

3^rd^-level errors, *t*-Test comparison

*t*(36) = - 2.06, *p* = 0.047, *d* = - 0.67
